# Wide-field imaging of birefringent synovial fluid crystals using lens-free polarized microscopy for gout diagnosis

**DOI:** 10.1038/srep28793

**Published:** 2016-06-30

**Authors:** Yibo Zhang, Seung Yoon Celine Lee, Yun Zhang, Daniel Furst, John Fitzgerald, Aydogan Ozcan

**Affiliations:** 1 Electrical Engineering Department, University of California, Los Angeles, CA, 90095, USA; 2 Bioengineering Department, University of California, Los Angeles, CA, 90095, USA; 3 California Nanosystems Institute, University of California, Los Angeles, CA, 90095, USA; 4 Division of Rheumatology, Department of Internal Medicine, University of California, Los Angeles, CA, 90095, USA; 5 Department of Surgery, David Geffen School of Medicine, University of California, Los Angeles, CA, 90095, USA

## Abstract

Gout is a form of crystal arthropathy where monosodium urate (MSU) crystals deposit and elicit inflammation in a joint. Diagnosis of gout relies on identification of MSU crystals under a compensated polarized light microscope (CPLM) in synovial fluid aspirated from the patient’s joint. The detection of MSU crystals by optical microscopy is enhanced by their birefringent properties. However, CPLM partially suffers from the high-cost and bulkiness of conventional lens-based microscopy, and its relatively small field-of-view (FOV) limits the efficiency and accuracy of gout diagnosis. Here we present a lens-free polarized microscope which adopts a novel differential and angle-mismatched polarizing optical design achieving wide-field and high-resolution holographic imaging of birefringent objects with a color contrast similar to that of a standard CPLM. The performance of this computational polarization microscope is validated by imaging MSU crystals made from a gout patient’s tophus and steroid crystals used as negative control. This lens-free polarized microscope, with its wide FOV (>20 mm^2^), cost-effectiveness and field-portability, can significantly improve the efficiency and accuracy of gout diagnosis, reduce costs, and can be deployed even at the point-of-care and in resource-limited clinical settings.

Gout is a type of crystal arthropathy, which is caused by the deposition of monosodium urate (MSU) crystals in the joints and periarticular structures such as the tendons and ligaments. During an acute gout attack, the patient experiences severe pain and swelling of the affected structures, which can often be debilitating for the patient^1^,^2^. The prevalence of gout has been gradually increasing by as much as fourfold for the past five decades, and is the most common type of inflammatory arthritis in the United States affecting over 8 million adults, 3.9% of the entire population^3^.

The pathogenesis of gout is complex, involving abnormalities in both metabolism and immunity. The key components include hyperuricemia and MSU crystallization^4^. Uric acid is a byproduct of purine metabolism, degraded by the enzyme uricase by most mammals; however, humans lack this enzyme because of multiple evolutional mutations of its coding gene and hence have higher levels of serum urate than other mammals^5^. Once serum urate rises above 6.8 mg/dL, urate can form MSU crystals under certain environmental factors (typically in and around joints), which then act as a potent trigger of inflammation in the joints^6^,^7^.

The gold standard for the diagnosis of gout is identification of MSU crystals within the synovial fluid aspirated from the joints of a gout patient using a compensated polarized light microscope (CPLM)^8^,^9^. Compared to a standard brightfield light microscope, a CPLM has a pair of linear polarizers using the cross-polarized configuration, and a full-wave retardation plate (red compensator) to convert birefringence of the objects into color variations. MSU crystals have needle-like shape and strong negative birefringence, i.e., the fast axis is along the axial direction of the crystal, which, when observed under a CPLM, appear *yellow* (or *blue*) when the MSU crystal is aligned *parallel* (or *perpendicular*) with the slow axis of the full-wave retardation plate, upon a red/magenta background color. Although polarized microscopy has been considered as the “gold standard” for diagnosis of gout since 1961, recent studies show that joint aspiration is not regularly performed in primary care clinics^10^,^11^. In some observational studies, only about 10% of primary care physicians performed polarizing microscope examination in diagnosing gout patients^12^,^13^. Among other reasons, limitations of traditional CPLM play an important role. Most critically, conventional lens-based microscopes have relatively small FOV, especially when high-numerical aperture (NA) and high-magnification objective lenses are used. For example, in the identification of MSU crystals, routinely a 40× (e.g., 0.75NA) objective lens is used to observe the morphology of the crystals, resulting in an extremely small FOV (~0.2 mm^2^) which leads to long examination times by diagnosticians. In particular, when there is only a limited number of crystals present in a synovial fluid sample taken from the patient, the examination of the entire sample can be not only time-consuming but also can produce a non-reliable diagnostic result because of operator-dependent bias in detecting the crystals over a limited FOV^14^. The concentration of crystals directly correlates with diagnosticians’ ability to positively identify crystals^15^. Furthermore, the reliability of CPLM for detection of MSU crystals can vary widely depending on the examiner’s level of training^16^. These drawbacks of the current method call for a newer method to detect birefringent crystals that is higher-throughput, easier to use and ideally automated.

Invented to address the limitations of the conventional lens-based light microscopes, lens-free on-chip microscopy based on digital in-line holography has gone through significant developments in the past decade^17^,^18^,^19^,^20^,^21^,^22^,^23^,^24^,^25^,^26^,^27^,^28^,^29^,^30^,^31^,^32^,^33^,^34^,^35^,^36^,^37^,^38^,^39^. Taking advantage of the rapid increase in mega pixel counts and the reducing costs of optoelectronic image sensors as well as the exponential improvements in computation capabilities of consumer electronic devices, lens-free on-chip microscopy can perform wide-field imaging without the need for lenses or objectives in a compact, cost-effective and field-portable setup^30^,^40^,^41^. In a lens-free on-chip imaging set-up, a transparent sample is positioned above an image sensor chip (e.g., a complementary metal-oxide semiconductor (CMOS) or charged-coupled device (CCD) chip) where the sensor-to-sample distance (i.e., *z*_2_) is less than a millimeter whereas the source-to-sample distance (*z*_1_) is e.g., 10–15 cm. This imaging geometry has unit magnification and as a result of this, the entire active area of the imager chip serves as the object FOV, which can easily reach >20–30 mm^2^ and >10–20 cm^2^, using commercially available CMOS or CCD imager chips, respectively. Therefore the FOV of the lens-free microscope can be 2–3 orders of magnitude larger than a conventional lens-based microscope with a similar resolution level^35^. One disadvantage of unit magnification geometry is that the diffraction patterns or in-line holograms of samples are typically undersampled due to the relatively large pixel size at the sensor plane. This limitation has been mitigated using pixel super-resolution (PSR) and synthetic aperture techniques to achieve submicron resolution over large FOVs, breaking the resolution limit imposed by the pixel size of the image sensor chips, also achieving a space-bandwidth product that is larger than 1 billion^21^,^23^,^35^,^42^,^43^,^44^,^45^,^46^,^47^.

Due to these advantages, lens-free computational microscopy can be a potential solution to the efficiency and reliability issues of gout diagnosis using the conventional CPLM. However, the adaptation of the current bright-field lens-free microscopy setup to polarized imaging is not straightforward: the cross-polarized configuration used in CPLM can totally extinct the background light that is not modified by the birefringent sample, and therefore is not applicable to lens-free holography where reference light is necessary to form interference^41^,^48^. Moreover, the color contrast of birefringent objects as generated by a conventional CPLM is challenging to replicate by a lens-free microscope which inherently uses narrow-band illumination sources, unless multiple wavelengths are used.

To face these challenges and create a lens-free microscope that can be used in gout diagnosis, we designed a novel *lens-free differential holographic polarized imaging* platform, which can achieve wide-field imaging of birefringent objects on an image sensor chip with sub-micron resolution (see Fig. 1(a)). In this design, a partially coherent light source is passed through a circular polarizer and is incident on the transparent sample that contains the synovial fluid taken from a patient. A CMOS optoelectronic image sensor is placed right below the sample, with a polarization analyzer unit composed of a λ/4 retarder film and a linear polarizer film positioned above the image sensor, which utilizes the entire active area of the image sensor as our imaging FOV (~20.5 mm^2^). The holographic diffraction patterns of the sample captured by the image sensor are processed by reconstruction algorithms including PSR^17^,^21^,^22^,^29^,^30^,^40^,^49^ and multi-height based phase recovery^22^,^29^,^30^,^40^ which generate both amplitude and phase images of the sample. In this work, we also employed a differential imaging strategy where we subtract two reconstructed images (where the analyzer was rotated by 90° between each image acquisition) to eliminate possible ambiguities between birefringent objects and absorptive objects that might randomly appear in the sample FOV. The detailed polarization design of our lens-free on-chip microscope is also shown in Fig. 1(b). The analyzer unit is modified from a standard cross-polarized configuration by changing the orientation of the linear polarizer from +45° with respect to the *x*-axis to +65°, to pass a certain fraction of the background light for hologram formation.

We optimized this lens-free polarization imaging design first using numerical simulations based on the Jones calculus^50^, and then experimentally validated its effectiveness by imaging MSU crystal samples made from a gout patient’s tophus and a fabricated steroid crystal sample (used as a negative control). Our lens-free polarized imaging results, after a digital pseudo-coloring step, achieved close agreement to gold-standard images obtained using CPLM with a 40× 0.75NA objective lens, both in color contrast and spatial details of the samples. Three board-certified rheumatologists also confirmed the image quality and the resolution of the lens-free polarized microscope images for crystal identification in gout diagnosis. With a substantial increase in the imaging FOV coupled with high resolution and contrast, this computational imaging technique is a promising approach to increase the efficiency and accuracy of gout diagnosis. This system can also potentially be used for pseudogout diagnosis, with the causative calcium pyrophosphate dihydrate (CPPD) crystals having positive-birefringence and rhomboid or rod shape. We believe that with its cost-effectiveness, compactness and field-portability, this lens-free polarization imaging technology shows great promise to be deployed at point-of-care and low-resource settings for gout disease and related clinical needs. Other future clinical applications of this platform could include diseases caused by crystals that can be detected by a conventional CPLM; for example, ureteral stones can be diagnosed by detecting birefringent crystals in urine samples.

## Methods

### Lens-free polarized on-chip imaging setup

**Figure 2:**
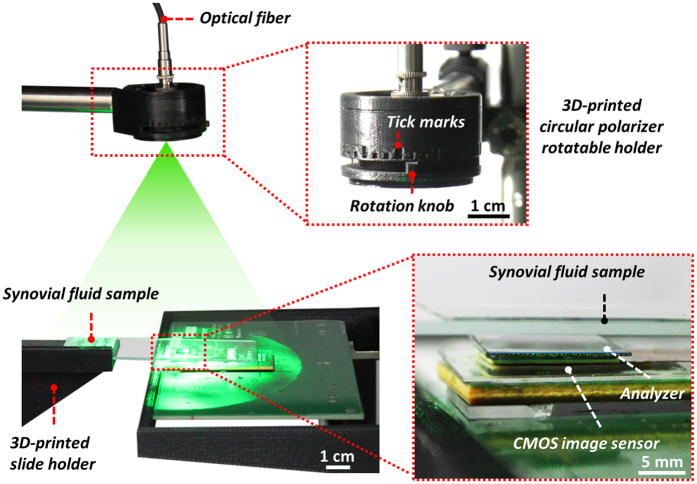
Experimental setup.

A broad band source (WhiteLase-Micro, Fianium Ltd, Southampton, UK) is used to provide illumination at a wavelength of 532 nm, with a spectral bandwidth of ~2.5 nm and an optical power of ~ 20 μW. The source is coupled to a single-mode optical fiber and the light is emitted at the end of this fiber without any collimation, as shown in Fig. 2. A circular polarizer mounted in a 3D-printed rotatable holder is attached to the optical fiber, such that the light first passes through the circular polarizer. Approximately 10 cm (*z*_*1*_ distance) under the illumination fiber tip, a microscope slide with a drop of synovial fluid (dried) is held in place by a 3D-printed slide holder. A CMOS image sensor (Sony, IMX081, 1.12 μm pixel size) with an analyzer film on top is placed under the sample and is connected to a 3D positioning stage (Thorlabs, NanoMax 606) for x-y-z movement to achieve PSR and multi-height based phase recovery. The analyzer film is directly placed on top of the image sensor with immersion oil in between. The function of the immersion oil is to mitigate interference fringes caused by the thin air gap between the analyzer and the image sensor surfaces. The distance between the CMOS image sensor photosensitive layer and the sample (*z*_*2*_ distance) is ~600 μm.

Before image acquisition, the orientation of the circular polarizer is rotated manually to maximize the total illumination power on the sample by observing the histogram of the live readout from the image sensor. This alignment step does not need to be repeated for further imaging experiments if the illumination part remains unchanged. For an unpolarized light source, no such alignment is necessary.

At the first stage of the image acquisition, the long side of the analyzer unit is aligned with the long side (i.e., horizontal direction) of the image sensor chip. After PSR and multi-height hologram acquisition, we rotate the analyzer unit by 90°, and repeat the same PSR and multi-height hologram acquisition process. Since the rotation of the analyzer is equivalent to the rotation of the sample, in an alternative design, one can permanently bond the analyzer unit to the image sensor chip and rotate the sample between the two imaging runs.

### Fabrication of the analyzer unit using low-cost polymeric polarizing and retardation films

A 1.8 cm-by-1.5 cm piece of λ/4 retarder film (75 μm thickness, Edmund Optics) is cut out from a larger sheet, with the long side parallel to the slow axis. A piece of linear polarizing film (180 μm thickness, Edmund Optics) of the same dimensions is cut, with the long side at −65° with respect to the polarization direction. Then the two pieces are aligned and bonded together using ultraviolet (UV)-curable adhesive (NOA 68, Norland Products, Cranbury, NJ) with the λ/4 retarder on top, and cured under a UV lamp.

### Fabrication of the circular polarizing unit

In general for an unpolarized light source, a piece of circular polarizer placed in front of the light source is sufficient to generate circularly polarized light, without the need for alignment. However, in our experimental set-up the light generated by the tunable illumination source is close to linearly polarized light. Therefore, the orientation of the circular polarizer in our set-up translates into output light intensity variations. To better utilize the power of the illumination source, we designed a 3D-printed rotatable holder for the circular polarizer to achieve free manual rotation with a range of 180°. First, a circular polarizer piece is cut from a larger sheet (left-handed plastic circular polarizer, Edmund Optics), and is glued to a 3D-printed rotary piece with a handle. Then the rotary piece is placed inside a 3D-printed outer shell with openings on the top and at the bottom, and with tick marks for 10° increments. Finally an optical fiber holder is embedded inside the same outer shell, on top of the rotary piece.

### Processing of lens-free polarized images

**Figure 3:**
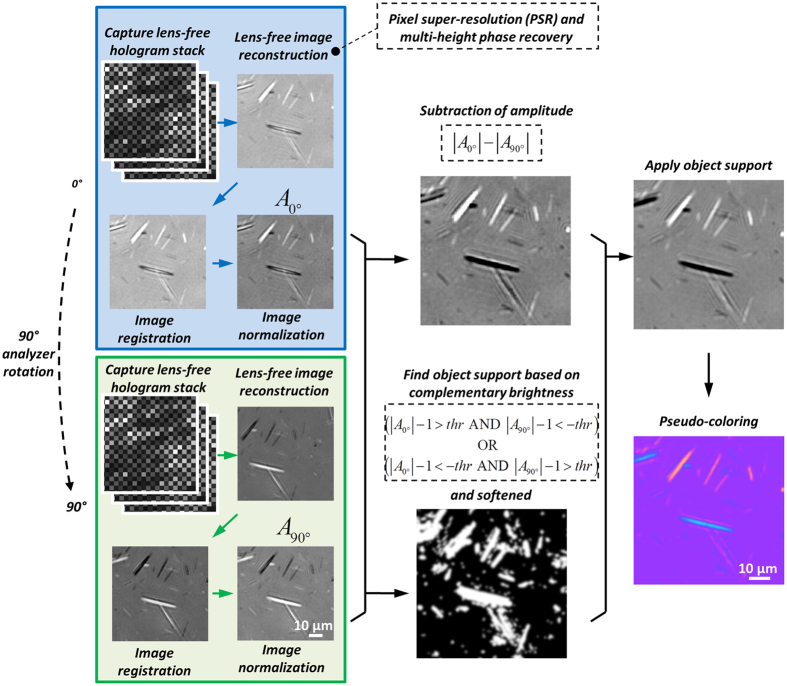
Image processing flow chart. The blue box and the green box depict the processing of the lens-free hologram stack with the analyzer positioned at 0° and 90°, respectively. Then the image processing results are used in combination to obtain the amplitude-subtracted (differential) image and the object support. Finally the object support is applied to obtain a grayscale image, and pseudo-coloring is performed to create color contrast similar to a CPLM.

As depicted in Fig. 3, after PSR and multi-height-based phase recovery and image reconstruction of the two hologram stacks with the analyzer unit undergoing a 90° rotation in between, two sets of reconstructed complex images of the objects are obtained. In order to combine them into a single lens-free polarized image with pseudo color-contrast, the following steps are sequentially applied:

#### Image registration

We utilize the automated feature-matching algorithm in the Computer Vision System Toolbox of MATLAB to calculate a geometric transform between the two sets of complex images assuming a similarity relationship, based on which the 90° image is aligned to the 0° image. Note that this feature matching requires that the inputs are real-valued images. Therefore, we used the absolute-background-subtracted versions of the two complex images for feature extraction purposes:


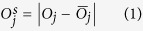


where *O*_*j*_ (*j *= {0°, 90°}) denotes the two complex images to be aligned, 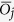 denotes the mean value of *O*_*j*_.

#### Image normalization

Both the 0° and 90° complex images after image registration in the previous step are divided by their respective mean values, such that the discrepancy between their brightness is minimized. This step results in two normalized complex images 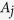 (*j* = {0°, 90°}).

#### Subtraction of image amplitudes

We then calculate 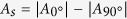, resulting in a differential image *A*_*s*_ whose values are centered around 0.

#### Birefringent object support calculation

To further exploit the information about the object support, i.e., the specific positions and maps of birefringent objects within the sample FOV, we take advantage of the complementary brightness property of this optical design, where *the brighter-than-background pixels caused by birefringence in the 0° image will roughly correspond to darker-than-background pixels in the 90° image*, and vice versa. Based on this, the object support map (*M*) for birefringent objects in our imaging FOV can be calculated using the following binary operation:


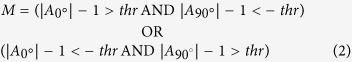


where *thr* is a predefined threshold value, e.g., 0.1, AND and OR refer to pixel-wise logical operators. The object support mask is then softened using a Gaussian function with *σ* = 0.56 μm, resulting in a new mask:


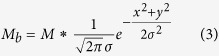


where * denotes two-dimensional convolution operation.

#### Application of object support

After the calculation of the birefringent object support map *M*_*b*_, we then create a grayscale differential image 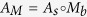, where 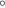 denotes pixel-wise multiplication of two images.

#### Pseudo-coloring of the lens-free image

In this final step, the grayscale differential image 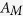 is mapped into a color image *C* to create a similar color contrast compared to a conventional CPLM image for the ease of a rheumatologist to inspect our lens-free images. This color map is statistically learned using a sample lens-free grayscale differential image from the previous step and a corresponding CPLM image (40× 0.75NA) of the same sample. First these two images (lens-free and CPLM) are aligned with respect to each other using image registration. Then, a set of 128 bins are created for the sample lens-free grayscale differential image, spanning the entire range of its values:


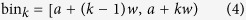


where *k = 1, …, 128*, *a* is the minimum value of the sample lens-free grayscale differential image (*A*_*M*_), and *a* + *128w* is equal to the maximum value of *A*_*M*_. For each one of these bins, we then perform the following:

- Find the set of pixels in the sample lens-free grayscale differential image that fall into the *k*th bin.
- For this set of pixels found in step a, find the corresponding pixels in the sample CPLM image, and calculate the mean R, G and B values for these pixels.

After steps a and b, we create the mapping between the pixel values of the sample lens-free differential image with respect to the R, G and B components of the corresponding CPLM image. We finally use a piecewise linear function to approximate these 3 mapping functions (for R, G and B channels) to avoid rapid fluctuations due to insufficient sampling. For values that can potentially occur outside the range of these bins, linear extrapolation method is used.

### PSR technique to improve the resolution of lens-free on-chip microscopy

The pixel size of the image sensor array imposes a physical limit on the resolution of a lens-free on-chip microscope, according to the Nyquist sampling theorem^51^. The PSR technique is applied to break this undersampling related resolution limit by capturing multiple subpixel-shifted low-resolution holograms and synthesizing them into a single high-resolution hologram^17^,^21^,^22^,^29^,^30^,^35^,^40^,^49^. During the lens-free hologram acquisition, a positioning stage is used to shift the image sensor chip on an 8-by-8 orthogonal grid with a grid size of 0.28 μm. Note that these subpixel shifts do not need to be precise or known a priori, as we use a digital shift estimation algorithm to accurately estimate these sub-pixel shifts after image capture^17^. Then a conjugate gradient method is used to find the optimal high-resolution hologram that is statistically consistent with all the low-resolution pixelated holograms that are undersampled at the sensor array^17^.

### Digital propagation of an optical wavefront using the angular spectrum method

If the complex wavefront of an optical field is known, which includes its amplitude and phase information, we can digitally calculate its propagation for a given distance using the angular spectrum method^48^. The complex field is first Fourier-transformed to the angular spectrum domain using a fast Fourier transform (FFT) algorithm. Then an optical phase function is calculated, parameterized by the wavelength, index of refraction of the medium, and the distance of the digital propagation. The multiplication of the angular spectrum of the original optical field and the calculated phase function is inverse Fourier transformed to the spatial domain, yielding the digitally propagated complex optical field.

### Autofocus algorithm to identify the sample height on the sensor chip

An autofocus algorithm is used to automatically find the *z*_*2*_ distance (i.e., the sample-to-sensor distance) for a PSR hologram by solving a maximization problem, with the objective function being a focus criterion, and the variable being the propagation distance. The focus criterion we used in this work is the negative of the Tamura coefficient^52^ calculated for the amplitude of the complex image, which is found to give a distinct peak at the correct *z*_*2*_distance. The hologram is digitally propagated to a range of *z*_*2*_ distances with the focus criterion evaluated at each height, and the corresponding maximum is found. Next, a smaller range of *z*_*2*_ distances are evaluated around this maximum point, with the scanning resolution also refined. These steps are repeated until the scanning resolution falls below a predefined threshold (e.g., 0.01 μm).

### Multi-height phase recovery for elimination of twin-image artifact

A multi-height iterative phase recovery algorithm^22^,^29^,^30^,^40^,^53^ with 10 heights is used to retrieve the optical phase of the holograms, in order to mitigate the twin image artifact caused by the loss of phase information at the sensor array. These heights are separated by ~15 μm. An initial guess of the complex optical wave is calculated using the back-propagation of the hologram at the first measurement height, assuming that the heights are ordered in ascending order (i.e., the closest *z*_*2*_ corresponds to the first height). Then, this initial guess is propagated to the second height, where its amplitude is averaged with the square root of the measured hologram at the second height, and the phase is kept unchanged. Next, this updating process is repeated at the subsequent heights and then backwards after it reaches the last height. Each one of these digital round-trips among these different heights counts as one iteration, and after ~10–20 iterations the optical phase converges, yielding us a unique complex wave for each one of the measurement heights. The converged complex wave of any one of these heights is finally propagated to the plane of the sample to obtain the complex image of the sample. Note that the transport of intensity equation (TIE)^22^,^54^,^55^ is not used here as it is known that TIE is more sensitive to low-frequency components, whereas the multi-height based iterative phase recovery is more sensitive to high-frequency components. In this work, since the birefringent crystals of interest in synovial fluid are relatively small and sharp, the multi-height iterative phase recovery converges rather quickly without the need for using a solution of TIE.

### Preparation of MSU and steroid crystals

The reference slides containing MSU crystals were anonymously prepared from a surgically resected large tophus without a link to any subject related information. The tophus was obtained when a patient with confirmed gout received resection surgery of the tophus located in the olecranon bursa. The surgery was routine elective surgery to alleviate the symptom, as part of standard clinical care and unrelated to this study. The tophus was cut in half, revealing a soft semi-liquid center. A smear sample was prepared (touch-prep method), and a small amount of adhesive mounting medium (Cytoseal™, Richard Allan Scientific, Kalamazoo, Michigan) was applied onto the sample. Finally, the slide was cover-slipped.

For the slides of steroid crystals (used as negative controls), a mixture of methylprednisolone acetate suspension (Depo-Medrol^®^ 40 mg/ml, Pfizer, New York) and 1 cc of 1% lidocaine was made. Twenty microliters of this mixture was placed onto a slide and smeared, and then air-dried. Adhesive mounting medium was not used for the steroid crystals slides, because applying the medium to steroid crystals had a tendency of creating bubbles next to the crystals, which was not observed in the MSU sample preparation.

All biologic samples were obtained after de-identifying the patients’ information. The methodology for obtaining these samples was reviewed by UCLA Institutional Review Board (IRB) and deemed exempt.

## Results

### Design, numerical simulation and analysis of lens-free polarized on-chip microscopy for imaging birefringent crystals

In order to model our optical design, we can effectively decompose the presented lens-free polarized imaging system into two sections that deal with *polarization* and *diffraction*. In the *polarization* related part, the circular polarizer, birefringent sample and the analyzer are assumed to be thin and the vertical gaps between these components are assumed to be negligible. In the *diffraction* part, the light that exits the *analyzer* diffracts to be sampled by the sensor chip, after a propagation distance of *z*_*2*_.

We formulated the polarization part of this lens-free on-chip imaging system using Jones calculus^50^ and simulated it in MATLAB. The Jones representation of the respective elements of our imaging system can be written as:

(a) Input left-hand circularly polarized (LHCP) light:


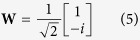


where 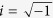.

Particular attention should be paid to the convention of handedness: the LHCP used in this paper is defined *from the point of view of the source*, i.e., *if one looks away from the source, along the direction of light propagation, the temporal rotation of the field at a given point in space is counterclockwise*.

(b) Birefringent sample:


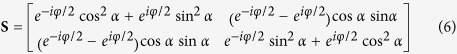


where 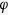 is the relative phase retardation induced by the object birefringence after the sample plane, and 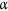 is the orientation of the fast axis of the birefringent sample with respect to the *x*-axis.

(c) λ/4 retarder:


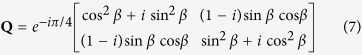


where 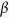 is the orientation of the fast axis of the λ/4 retarder with respect to the *x*-axis.

(d) Linear polarizer:


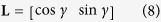


where 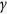 is the polarization orientation of the linear polarizer with respect to the *x*-axis. Note that we write **L** as a row vector instead of a 2-by-2 matrix, such that *p *= **LQSW** can be a scalar complex output.

Based on these definitions, the variables of interest in our lens-free optical design for polarization imaging are *α, β, γ*, and *φ*. In our simulations, we approximated the shape of the MSU crystals as a cylinder. We further assumed that the lower bound on the diameter of the MSU crystal is 0.5 μm^56^, and the lower bound on the birefringence is 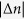 = 0.1 with the fast axis being the axis of the cylinder; therefore the relative birefringence induced phase retardation at the center of the cylinder at a wavelength of 532 nm can be approximated as 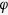 ~ 0.19π. As the incident wave is circularly polarized, without loss of generality, we select *β* to be equal to 90°. In order to detect birefringence as well as its sign (+/−) similar to a CPLM image, ideally the brightness in the output image should vary when the MSU crystal takes different orientations in the sample FOV. More specifically, when the MSU crystals are aligned with a certain direction, the output should appear brighter than the background; when perpendicular to the same direction, the output should appear darker than the background. In this way, if the sign of the birefringence changes, the brightness variation will invert, helping us to determine the sign of the birefringence of the sample.

**Figure 4:**
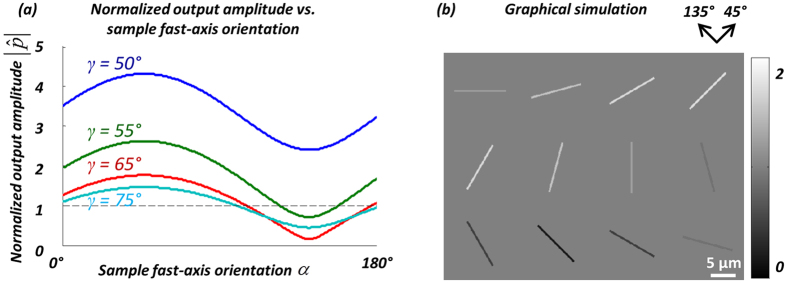
(**a**) Optimization of the orientation angle of the linear polarizer (*γ*). The normalized output amplitude 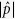 is plotted as a function of the sample fast-axis orientation, *α*, for different linear polarizer orientations (*γ = *50°, 55°, 65°, 75°). (**b**) The simulated normalized output images of MSU crystals at varying orientations, using *γ *= 65°. The MSU crystals are simulated as cylinders with a birefringence of *|*Δ*n| *= 0.1, diameter of 0.5 μm, length of 10 μm, and the fast axis is along the long axis of the crystals.

With these in mind, we scanned the remaining two parameters *α* and *γ*, and calculated the normalized output (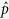) against *α* while varying *γ*, i.e.:


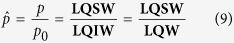


where in the calculation of *p*_*0*_, the Jones matrix of the birefringent sample is replaced by the identity matrix **I** representing no sample being present. As can be seen in Fig. 4(a), all the curves corresponding to different choices of *γ* exhibit a modulation of 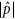 as a function of *α*, and the maximum values of these curves occur at *α* = 45° while the minimum values occur at *α* = 135°. Among all of these curves shown in Fig. 4(a), the red curve, representing *γ *= +65°, has the largest modulation depth, implying the best sensitivity for the current parameters simulated. Moreover, the red curve is almost symmetrically distributed around unity, and thus, the brighter-than-background orientations of the MSU crystal roughly correspond to 0° < *α* < 90°, whereas the darker-than-background orientations of the MSU crystal roughly correspond to 90° < *α* < 180°. This feature gives advantage to the determination of the sign of the birefringence of the objects which is important for gout diagnosis and inspection of synovial fluids, and therefore in our experimental design, we chose *γ* = +65° as the optimal configuration. Based on this choice, Fig. 4(b) also shows the graphical simulation of the image of a 0.5 μm diameter MSU crystal having different orientations: as expected, the crystal brightness is maximum when aligned in the 45° direction, and minimum when aligned in the 135° direction.

Next, we further simulated the behavior of four different types of objects with the same cylindrical morphology with a diameter of 0.5 μm:

1. Transparent and negatively birefringent (*φ *= 0.19π, fast axis is along the cylinder axis);
2. Transparent and positively birefringent (*φ *= 0.19π, fast axis is perpendicular to the cylinder axis);
3. Transparent and non-birefringent (*φ *= 0);
4. Absorptive and non-birefringent (*φ *= 0 and transmission light intensity is attenuated by 36% per micron).

**Figure 5:**
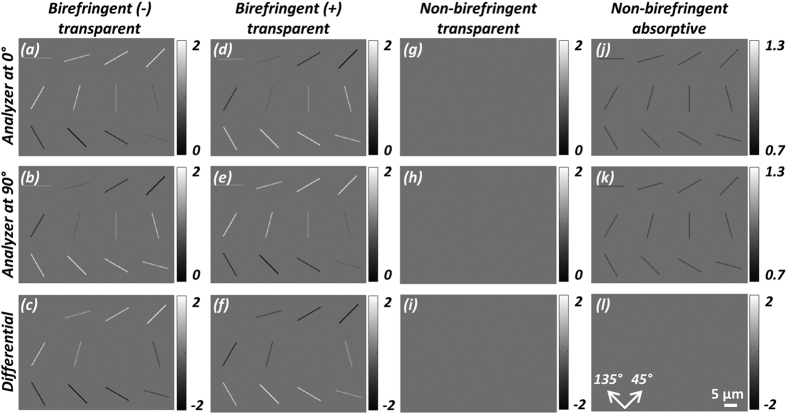
Simulated images of four different types of particles with the same needle-like morphology: negatively birefringent and transparent (first column), positively birefringent and transparent (second column), non-birefringent and transparent (third column), non-birefringent and absorptive (fourth column), imaged under two different analyzer orientations (0°: first row, 90°: second row) and the subtraction of the amplitudes (labeled as *differential*) at these two orientations (third row). The differential step (third row) results in cancellation of non-birefringent particles that normally appear in both orientations of the analyzer.

These numerical simulations were performed to better understand how different target objects would appear in our imaging design as compared to potential false positive objects, and the results are summarized in Fig. 5. As can be seen in the first row, Fig. 5(a,d), having opposite signs of birefringence show inversion of brightness; for example for *α* = 45°, negative birefringence translates to maximum brightness while positive birefringence translates to minimum brightness; for *α* = 135°, negative birefringence translates to minimum brightness while positive birefringence translates to maximum brightness. As expected, a non-birefringent and transparent object (see Fig. 5(g)) results in zero signal, whereas a non-birefringent and absorptive object (see Fig. 5(j)) results in reduced brightness.

A close observation of Fig. 5 (panels a,d, and j) reveals a potential ambiguity of crystal analysis. Although it is safe to declare brighter-than-background objects as birefringent, darker-than-background objects need additional analysis before they can be described as birefringent since an absorptive object could have the same appearance upon single viewing. To resolve this ambiguity, we adopted a differential imaging strategy as also detailed in the Methods section. In addition to a single analyzer/sample orientation, we rotate the analyzer or the sample by 90°, then repeat the lens-free imaging experiment, and finally subtract the amplitudes of the two reconstructed images, resulting in the differential output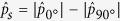, where the subscripts 0° and 90° denote the images before and after analyzer/sample rotation, respectively. Figure 5 middle row depicts the second set of reconstructed images with the analyzer rotated by 90°, and Fig. 5 bottom row shows the subtraction results. *As shown in* Fig. 5(c,f,l)*, the signals due to birefringence are enhanced while the signals due to absorption are exactly canceled out, as desired*. This differential image 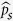, in combination with the original lens-free images, 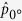
and 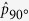, help us remove potential false positive objects while also sensitively detecting birefringent objects and determining their sign. One should note that if a specific birefringent crystal is aligned either at 0° or 90°, the difference lens-free image
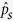
will not show its signature; this is also the case for the standard CPLM and would not constitute a limitation since the individual images at each analyzer position will show the presence of such birefringent crystals (see e.g., Fig. 5(a–f)).

**Figure 6:**
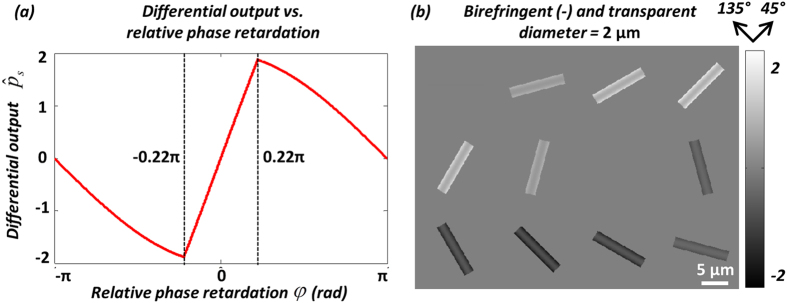
(**a**) Simulation of the differential output 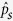 as a function of the relative phase retardation *φ*, with the crystals aligned at 45° (*α *= 45°). 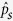 almost linearly reaches to the maximum/minimum when *|φ|* increases from 0 to ~ 0.22π, then turns backwards towards 0 as *|φ|* further increases to π. (**b**) Simulated image of a MSU crystal with larger diameter (2 μm) compared to Figs 4 and 5. The effect of the nonlinearity is manifested by the hollow appearance of the simulated images. Nevertheless, the intense (bright/dark) edges provide enough contrast for crystal detection and identification.

For this differential lens-free imaging design, it is also important to understand and quantify the linearity of the differential output signal 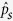 with respect to the relative birefringent phase retardation *φ*. Here, the crystals are assumed to be aligned at 45° (*α *= 45°). Since 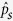
is a periodic function of *φ* with a period of 2π, we only need to investigate 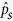 with respect to *φ* varying between −π and π, where 0 < *φ *< π implies that the fast axis is along 45°, and −π < *φ* *< *0 implies that the slow axis is along 45°. As shown in Fig. 6(a), for small *φ* (|*φ*| < 0.22π), the differential output 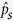 is almost perfectly linear as a function of *φ*. However this linearity does not hold for larger *|φ|*. In fact, beyond the turning points *|φ| *≈ 0.22π, the curve moves backwards and reaches zero at *|φ| *= π. This is an interesting observation that is revealed by our numerical simulations and analysis, and it should not affect the sensitivity of our imaging platform for gout diagnosis or detection of MSU crystals. The thickness of the needle-shaped MSU crystal gradually increases from its edge (approximately zero thickness) to the middle (largest thickness), so that the relative phase retardation *φ* also gradually increases from 0 to its maximum value. Therefore, it is guaranteed that even for a thick MSU crystal with a large maximum *φ* value, there will be a strong linear birefringence signal toward the edges of the crystal for its detection and identification. This is also verified by the simulation results shown in Fig. 6(b), where the diameter of the cylindrical crystal model is increased to 2 μm, and therefore the maximum relative phase retardation is increased to approximately 0.75π. It is shown that, even though at the middle of the crystals the images appear less intense, the strong signal contrast toward the edges is maintained. The same behavior is also verified experimentally, as will be detailed in the next subsection.

### Experimental results on lens-free polarized imaging of MSU crystals

**Figure 7:**
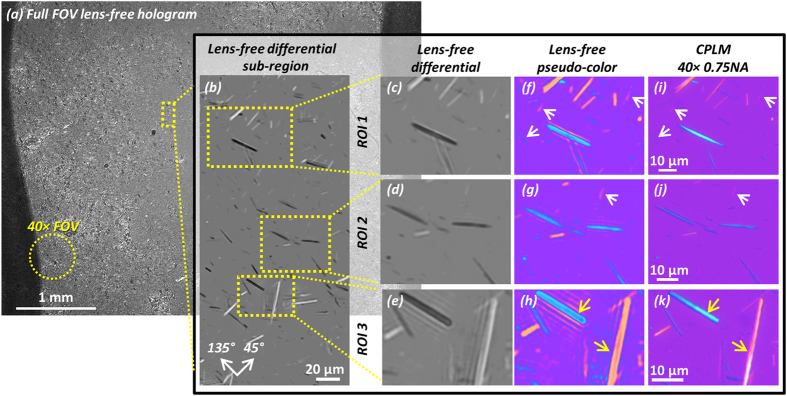
Experimental imaging result of a MSU crystal sample from a patient’s tophus, compared to a 40× 0.75NA CPLM. (**a**) The full FOV of the lens-free hologram is 20.5 mm^2^ which is ~2 orders of magnitude larger compared to the FOV of a typical 40× microscope objective lens (see yellow dashed circle). (**b**) A sub-region showing the lens-free differential polarized image. Clearly the crystals oriented close to 45° (see orientation guide in the bottom left) appear brighter than the background, and those close to 135° appear darker. (**c–e**) Lens-free grayscale differential image of 3 ROIs taken from (**b**). (**f–h**) Pseudo-colored images of (**c–e**). (**i–k**) 40× 0.75NA CPLM images of the same regions as (**f–h**). White arrows: crystals that result in a weak signature have better contrast in the lens-free pseudo-color images (**f,g**) than the CPLM images (**i,j**). Yellow arrows: thick MSU crystals in the lens-free pseudo-color image (**h**) have hollow appearances, slightly different from the CPLM image (**k**).

To demonstrate the imaging capabilities of our lens-free polarized on-chip microscopy platform to be used in gout diagnosis, we imaged MSU crystal samples made from the tophus of a de-identified patient (refer to the Methods section for details) using our lens-free microscope and compared our images against the gold standard images captured using a benchtop CPLM (Olympus BX51 with additional polarization components: drop-in polarizer U-POT and gout analyzer U-GAN) with a 40× 0.75NA objective lens. Figure 7(a) shows a full-FOV lens-free hologram, captured with the analyzer at 0°. The circular FOV of a typical 40× objective lens (see the yellow dashed circle) is ~0.24 mm^2^, which is around two orders of magnitude smaller compared to the lens-free FOV. This large FOV of lens-free microscopy offers an important advantage for screening of large areas in the search for scarce crystals, potentially helping to reduce the false-negative rate of diagnosticians. By digitally zooming into a sub-region of the lens-free image (see Fig. 7(b)), one can clearly see that, as expected, the MSU crystals appear brighter compared to the background when their orientations are close to 45° and darker when their orientations are close to 135°. Three regions of interest (ROI) are further selected and zoom-in images are shown to the right of Fig. 7. The lens-free pseudo-colored images (Fig. 7(f–h)) are digitally processed from the lens-free grayscale differential reconstruction results (Fig. 7(c–e)) as detailed in the Methods section. Comparing Fig. 7(f,g) to the corresponding images of the benchtop CPLM (Fig. 7(i,j)), we notice that not only the most prominent objects with strongly yellow or blue colors agree well in each set of images, but even the weak signals are picked up (pointed by the white arrows) by both microscopes; in fact the image contrast of these weak crystals captured by the lens-free microscope is much stronger than the CPLM images. This stronger image contrast suggests the potential enhanced sensitivity of the lens-free polarized microscope. In Fig. 7(h), we also notice that two relatively thicker crystals (pointed by the yellow arrows) result in “hollow” appearances, verifying the predictions of our numerical simulations (see Fig. 6(b)). Although Fig. 7(h) appears somewhat different compared to Fig. 7(k), it should not pose a problem for identification of MSU crystals or gout diagnosis, as these thick MSU crystals are clearly defined by their strong yellow/blue periphery enclosing a hollow interior, with a needle-shaped morphology. For the same thicker crystals, we also notice that the lens-free images contain some fringes along the crystals that do not exist in the traditional CPLM images. These artifacts result due to diffraction and form a signature of thicker birefringent crystals in lens-free images. However, because of the fact that these fringes will only occur around these thick and strongly birefringent objects and that non-birefringent objects (transparent or absorptive) are canceled out in our differential holographic images, this will not affect the sensitivity of our computational gout imaging method.

### Experimental results on lens-free polarized imaging of steroid crystals

**Figure 8:**
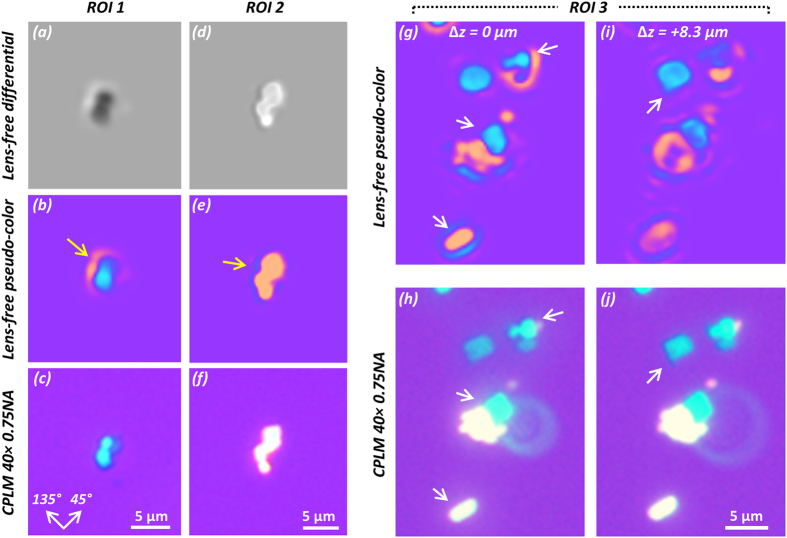
Experimental imaging result of a steroid crystal sample, compared to a 40× 0.75NA CPLM. (**a,d**) Lens-free grayscale differential images of ROI 1 and ROI 2. (**b,e**) Pseudo-colored images of (**a,d**). The yellow arrows point to the glowing effect around crystals, resulting from the large thicknesses of the crystal particles. (**c,f**) 40× 0.75NA CPLM images of the same regions as (**b,e**). (**g,i**) The lens-free images of ROI 3 digitally refocused to the best relative focus distances (Δ*z*) for different crystal particles, pointed by white arrows. (**h,j**) CPLM images corresponding to (**g,i**), manually refocused to the best focus distances for the respective particles pointed by white arrows.

Next, in order to test the performance of our lens-free holographic imaging method to differentiate other types of birefringent crystals from MSU crystals, we imaged steroid crystal samples as negative control. Corticosteroid crystals are birefringent crystals that can be found in some patients’ joint fluids following a corticosteroid injection and sometimes can lead to false positives in gout diagnosis. Their irregular shape provides a means to differentiate them from MSU crystals. As shown in Fig. 8, the pseudo-color lens-free polarized microscope images (b, e, g, i) of these crystals show consistent morphology that agrees well with the benchtop CPLM images of the same samples (c, f, h, j). Because of the large thicknesses of these steroid crystals, there exists some glowing artifacts around the crystals’ lens-free images (b, e, yellow arrows), due to similar reasons previously discussed. In particular, the ROI 3 shown in Fig. 8(g–j) contains multiple steroid crystal particles, whose surfaces reside at different depths/heights. We use the digital re-focusing capability of the lens-free polarized microscope to show some of the in-focus images of these respective crystal particles at different z-distances from the sensor chip. In Fig. 8(g,i), the lens-free image was digitally refocused to relative Δ*z* distances of 0 μm and 8.3 μm. At these respective planes, the particles on the lens-free images pointed by the white arrows are at the best focus, showing distinct and clear shapes that are also consistent with Fig. 8(h, j), which had to be manually refocused to the same particles due to the extremely narrow depth of focus of the objective lens used in CPLM. For example, the blue-colored irregularly shaped crystal particle on the top right of ROI 3, pointed by the white arrow in Fig. 8(g), is best visualized at Δ*z *= 0 μm, and the sharp corner at the bottom of the blue-colored crystal particle, pointed by the white arrow in Fig. 8(i), is best visualized at Δ*z *= 8.3 μm.

This digital re-focusing capability of the lens-free holographic polarized microscope is an important feature and an advantage for the diagnosis of gout since microscopic samples are usually not perfectly planar – they inevitably have height variations on the order of tens of microns. Moreover, when the user of a conventional microscope translates the sample stage to observe different regions of the sample, the sample can easily get out of focus as the movement of the sample stage is not perfectly horizontal. For a regular sample, since one can constantly refocus the microscope, these issues may be acceptable (at the cost of diagnostician time). But when screening a sample with scarce crystals using a standard benchtop CPLM, there can be scenarios where there are simply not enough birefringent targets to focus on^57^. This would be less of an issue for the lens-free holographic polarized microscope described in this work because of its enhanced depth of field which can span several hundred microns as well as its large FOV that is >20 mm^2^ 48. The lens-free holograms over a large sample area can thus be easily brought into focus by autofocusing and digital back-propagation algorithms detailed in our Methods section.

## Discussion

One of the major advantages of the presented lens-free polarization imaging approach is its larger FOV and cost-effectiveness compared to a standard CPLM. Using state-of-the-art CMOS imager chips that are also found in mobile phone cameras, a large FOV of >20–30 mm^2^ can be routinely achieved using the lens-free imaging technology, whereas a standard objective-lens with a similar resolution level would typically have ~0.2 mm^2^ FOV. Furthermore, a field-portable and light-weight version of our lens-free microscope with PSR capabilities^30^ can be put together under $250–300 including all the components except the computer interface, while a standard CPLM^58^ costs more than $10K and is significantly more bulky and heavy (>15 kg), also excluding the PC.

**Figure 9:**
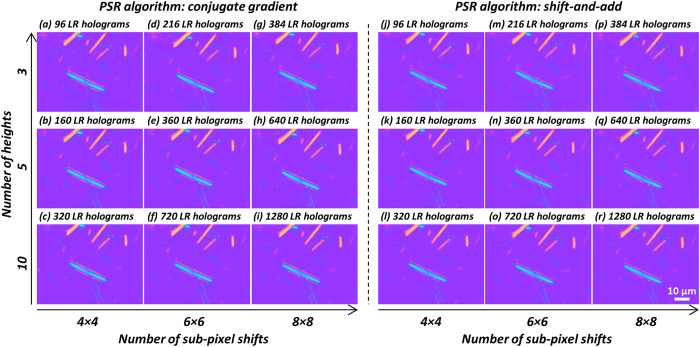
Comparison of lens-free polarized imaging results using different numbers of low-resolution (LR) raw holograms. The left panel uses the conjugate gradient algorithm for implementing PSR, while the right panel uses the shift-and-add algorithm. Columns: different numbers of sub-pixel shifted raw holograms are used to synthesize a high-resolution hologram using PSR. Rows: different numbers of heights are used to perform multi-height phase recovery. The total number of LR raw holograms used to generate each image also includes a factor of 2 due to the two analyzer orientations.

The imaging time of a large sample FOV is also of importance for this lens-free polarization microscopy technique to be used for synovial fluid screening in clinical settings. The image acquisition and processing speeds of the presented platform are currently not optimized to fully utilize the capabilities of the image sensor chip and the computer used in our work. In the image acquisition step, typically 1280 raw (i.e., lower-resolution) holograms are captured in each experiment for PSR (64 raw holograms per height), multi-height phase recovery (10 heights) and differential imaging (2 analyzer angles). However, a significant reduction in the number of raw holograms that need to be acquired can be achieved as illustrated in Fig. 9: a similar image quality is retained in our lens-free MSU crystal images by using e.g., 4× 4 = 16 raw holograms for PSR, 3 multi-height measurements at 2 analyzer angles, resulting in 96 raw holograms in total, which presents more than 13-fold reduction in the number of raw hologram measurements (96 vs. 1280). The CMOS image sensor chip that we used in our experiments has a maximum frame rate of ~15 frames per second; however, a much faster image acquisition can be achieved by adopting higher frame rate image sensors already available on the market.

Figure 9 also compares the performance of two different PSR algorithms (i.e., the conjugate gradient algorithm^30^,^40^,^43^ vs. the shift-and-add algorithm^21^,^22^,^44^) as a function of the number of raw holograms used in phase recovery and image reconstruction, both of which provide very similar imaging results. Using the shift-and-add algorithm for PSR, which is in general faster than the conjugate gradient algorithm, the entire image reconstruction of a 1 mm^2^ FOV for one analyzer orientation (including PSR with 64 sub-pixel shifts and multi-height phase recovery with 10 heights, same parameters as in Fig. 9(r)) can be completed within ~2.5 min using a desktop PC (OptiPlex 9010, Dell Inc.), without any GPU programming. The computation time cost of the subsequent image processing steps (image registration, image subtraction, pseudo-coloring, etc.) is much smaller compared to PSR and multi-height phase recovery steps. Therefore, for each analyzer orientation a computer cluster with approximately 20 nodes can process an entire FOV of ~20 mm^2^ in ~2.5 min by dividing it into 1 mm^2^ tiles. This image computation time for a given analyzer orientation is significantly reduced to ~20 seconds for PSR with 16 sub-pixel shifts and multi-height phase recovery using 3 heights, same as in Fig. 9(j). To further improve the image processing speed, a cluster of GPUs can be used instead of CPUs, which can speed up the reconstruction time by at least another factor of 10-fold.

## Conclusions

We presented the design of a lens-free differential holographic polarized imaging platform, which integrates cost-effective polymeric polarizing films with lens-free on-chip microscopy to achieve wide-field and high-resolution imaging of birefringent crystals. This computational imaging system is designed and further optimized through numerical simulations using Jones calculus. Lens-free imaging experiments corresponding to MSU crystal samples prepared from a gout patient’s tophus and steroid crystals (as negative control) demonstrated that our reconstructed images have resolution, color and contrast that are highly consistent with the performance of a gold-standard CPLM (40× 0.75NA). With ~2 orders of magnitude larger FOV than a CPLM, the presented technique has the potential to largely improve the efficiency and accuracy of gout diagnosis, while also reducing costs. Furthermore, as the lens-free imaging set-up can be cost-effective and field-portable, the presented method is especially promising for automated diagnosis of crystal arthropathy at the point of care or in resource-limited clinical settings.

## Additional Information

**How to cite this article**: Zhang, Y. *et al*. Wide-field imaging of birefringent synovial fluid crystals using lens-free polarized microscopy for gout diagnosis. *Sci. Rep*. **6**, 28793; doi: 10.1038/srep28793 (2016).

## Figures and Tables

**Figure 1:**
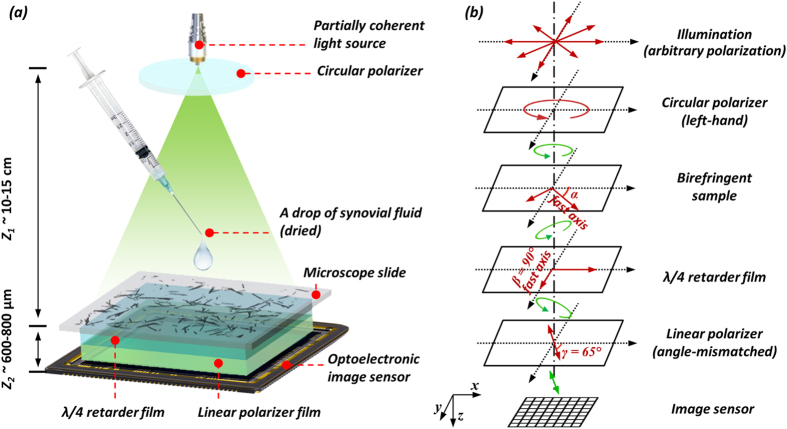
(**a**) Schematic setup of lens-free differential holographic polarized microscopy. (**b**) Design of the polarization in this system. The light, which is propagating from top to bottom, passes through a left-hand circular polarizer, the birefringent sample, a λ/4 retarder film, a linear polarizer and reaches the image sensor. The orientations of the polarizing components are illustrated with red arrows, and the polarization states of the light between components are illustrated with green arrows.

## References

[b1] Crystal-induced arthropathies: gout, pseudogout, and apatite-associated syndromes. (Taylor & Francis, 2006).

[b2] Neogi T. . 2015 Gout Classification Criteria: An American College of Rheumatology/European League Against Rheumatism Collaborative Initiative. Arthritis Rheumatol. 67, 2557–2568 (2015). 26352873 10.1002/art.39254 PMC4566153

[b3] Zhu Y., Pandya B. J. & Choi H. K. Prevalence of gout and hyperuricemia in the US general population: The National Health and Nutrition Examination Survey 2007–2008. Arthritis Rheum. 63, 3136–3141 (2011). 21800283 10.1002/art.30520

[b4] McGill N. W. & Dieppe P. A. Evidence for a promoter of urate crystal formation in gouty synovial fluid. Ann. Rheum. Dis. 50, 558–561 (1991). 1888197 10.1136/ard.50.8.558 PMC1004487

[b5] Oda M., Satta Y., Takenaka O. & Takahata N. Loss of Urate Oxidase Activity in Hominoids and its Evolutionary Implications. Mol. Biol. Evol. 19, 640–653 (2002). 11961098 10.1093/oxfordjournals.molbev.a004123

[b6] Fiddis R. W., Vlachos N. & Calvert P. D. Studies of urate crystallisation in relation to gout. Ann. Rheum. Dis. 42, 12–15 (1983). 6615025 10.1136/ard.42.suppl_1.12 PMC1035033

[b7] Sours R. E., Fink D. A. & Swift J. A. Dyeing Uric Acid Crystals with Methylene Blue. J. Am. Chem. Soc. 124, 8630–8636 (2002). 12121104 10.1021/ja026083w

[b8] McCarthy D. J. & Hollander J. L. Identification of urate crystals in gouty synovial fluid. Ann. Intern. Med. 54, 452–460 (1961). 13773775 10.7326/0003-4819-54-3-452

[b9] Pascual E., Batlle-Gualda E., Martínez A., Rosas J. & Vela P. Synovial Fluid Analysis for Diagnosis of Intercritical Gout. Ann. Intern. Med. 131, 756–759 (1999). 10577299 10.7326/0003-4819-131-10-199911160-00007

[b10] Pal B., Foxall M., Dysart T., Carey F. & Whittaker M. How is Gout Managed in Primary Care? A Review of Current Practice and Proposed Guidelines. Clin. Rheumatol. 19, 21–25 (2000). 10752494 10.1007/s100670050005

[b11] Harrold L. R. . Primary care providers’ knowledge, beliefs and treatment practices for gout: results of a physician questionnaire. Rheumatology 52, 1623–1629 (2013). 23620554 10.1093/rheumatology/ket158 PMC3741476

[b12] Underwood M. Diagnosis and management of gout. BMJ 332, 1315–1319 (2006). 16740561 10.1136/bmj.332.7553.1315 PMC1473078

[b13] Amer H., Swan A. & Dieppe P. The utilization of synovial fluid analysis in the UK. Rheumatology 40, 1060–1063 (2001). 11561120 10.1093/rheumatology/40.9.1060

[b14] Schumacher H. R. . Reproducibility of synovial fluid analyses. A study among four laboratories. Arthritis Rheum. 29, 770–774 (1986). 3718565 10.1002/art.1780290610

[b15] Gordon C., Swan A. & Dieppe P. Detection of crystals in synovial fluids by light microscopy: sensitivity and reliability. Ann. Rheum. Dis. 48, 737–742 (1989). 2478085 10.1136/ard.48.9.737 PMC1003866

[b16] Park J. W. . Clinical factors and treatment outcomes associated with failure in the detection of urate crystal in patients with acute gouty arthritis. Korean J. Intern. Med. 29, 361–369 (2014). 24851071 10.3904/kjim.2014.29.3.361 PMC4028526

[b17] Bishara W., Su T.-W., Coskun A. F. & Ozcan A. Lensfree on-chip microscopy over a wide field-of-view using pixel super-resolution. Opt. Express 18, 11181 (2010). 20588977 10.1364/OE.18.011181 PMC2898729

[b18] Seo S. . High-Throughput Lens-Free Blood Analysis on a Chip. Anal. Chem. 82, 4621–4627 (2010). 20450181 10.1021/ac1007915 PMC2892055

[b19] Isikman S. O. . Lens-free optical tomographic microscope with a large imaging volume on a chip. Proc. Natl. Acad. Sci. 108, 7296–7301 (2011). 21504943 10.1073/pnas.1015638108 PMC3088621

[b20] Kim S. B. . Lens-Free Imaging for Biological Applications. J. Lab. Autom. 17, 43–49 (2012). 22357607 10.1177/2211068211426695 PMC3685198

[b21] Greenbaum A. . Increased space-bandwidth product in pixel super-resolved lensfree on-chip microscopy. Sci. Rep. 3 (2013).

[b22] Greenbaum A. . Wide-field computational imaging of pathology slides using lens-free on-chip microscopy. Sci. Transl. Med. 6, 267ra175–267ra175 (2014). 10.1126/scitranslmed.3009850 25520396

[b23] Luo W., Greenbaum A., Zhang Y. & Ozcan A. Synthetic aperture-based on-chip microscopy. Light Sci. Appl. 4, e261 (2015).

[b24] McLeod E. . High-Throughput and Label-Free Single Nanoparticle Sizing Based on Time-Resolved On-Chip Microscopy. ACS Nano 9, 3265–3273 (2015). 25688665 10.1021/acsnano.5b00388

[b25] Mudanyali O., Oztoprak C., Tseng D., Erlinger A. & Ozcan A. Detection of waterborne parasites using field-portable and cost-effective lensfree microscopy. Lab. Chip 10, 2419 (2010). 20694255 10.1039/c004829a PMC2942761

[b26] Su T.-W., Erlinger A., Tseng D. & Ozcan A. Compact and Light-Weight Automated Semen Analysis Platform Using Lensfree on-Chip Microscopy. Anal. Chem. 82, 8307–8312 (2010). 20836503 10.1021/ac101845q PMC2987715

[b27] Tseng D. . Lensfree microscopy on a cellphone. Lab. Chip 10, 1787 (2010). 20445943 10.1039/c003477k PMC2941438

[b28] Mudanyali O., Bishara W. & Ozcan A. Lensfree super-resolution holographic microscopy using wetting films on a chip. Opt. Express 19, 17378 (2011). 21935102 10.1364/OE.19.017378 PMC3258299

[b29] Greenbaum A. & Ozcan A. Maskless imaging of dense samples using pixel super-resolution based multi-height lensfree on-chip microscopy. Opt. Express 20, 3129 (2012). 22330550 10.1364/OE.20.003129 PMC3364049

[b30] Greenbaum A., Sikora U. & Ozcan A. Field-portable wide-field microscopy of dense samples using multi-height pixel super-resolution based lensfree imaging. Lab. Chip 12, 1242 (2012). 22334329 10.1039/c2lc21072j

[b31] Isikman S. O., Bishara W. & Ozcan A. Lensfree On-chip Tomographic Microscopy Employing Multi-angle Illumination and Pixel Super-resolution. J. Vis. Exp, doi: 10.3791/4161(2012) PMC3487288 22929176

[b32] Isikman S. O., Greenbaum A., Luo W., Coskun A. F. & Ozcan A. Giga-Pixel Lensfree Holographic Microscopy and Tomography Using Color Image Sensors. PLoS ONE 7, e45044 (2012). 22984606 10.1371/journal.pone.0045044 PMC3440383

[b33] Su T.-W., Xue L. & Ozcan A. High-throughput lensfree 3D tracking of human sperms reveals rare statistics of helical trajectories. Proc. Natl. Acad. Sci. 109, 16018–16022 (2012). 22988076 10.1073/pnas.1212506109 PMC3479566

[b34] McLeod E., Luo W., Mudanyali O., Greenbaum A. & Ozcan A. Toward giga-pixel nanoscopy on a chip: a computational wide-field look at the nano-scale without the use of lenses. Lab. Chip 13, 2028–2035 (2013). 23592185 10.1039/c3lc50222h PMC3813829

[b35] Greenbaum A. . Imaging without lenses: achievements and remaining challenges of wide-field on-chip microscopy. Nat. Methods 9, 889–895 (2012). 22936170 10.1038/nmeth.2114 PMC3477589

[b36] Weidling J., Isikman S. O., Greenbaum A., Ozcan A. & Botvinick E. Lens-free computational imaging of capillary morphogenesis within three-dimensional substrates. J. Biomed. Opt. 17, 126018–126018 (2012). 23235893 10.1117/1.JBO.17.12.126018 PMC3521054

[b37] Stybayeva G. . Lensfree Holographic Imaging of Antibody Microarrays for High-Throughput Detection of Leukocyte Numbers and Function. Anal. Chem. 82, 3736–3744 (2010). 20359168 10.1021/ac100142a PMC2864520

[b38] Kesavan S. V. . High-throughput monitoring of major cell functions by means of lensfree video microscopy. Sci. Rep. 4 (2014). 10.1038/srep05942 PMC5380008 25096726

[b39] Vashist S. K., Luppa P. B., Yeo L. Y., Ozcan A. & Luong J. H. T. Emerging Technologies for Next-Generation Point-of-Care Testing. Trends Biotechnol. 33, 692–705 (2015). 26463722 10.1016/j.tibtech.2015.09.001

[b40] Greenbaum A., Akbari N., Feizi A., Luo W. & Ozcan A. Field-Portable Pixel Super-Resolution Colour Microscope. PLoS ONE 8, e76475 (2013). 24086742 10.1371/journal.pone.0076475 PMC3785454

[b41] Mudanyali O. . Compact, light-weight and cost-effective microscope based on lensless incoherent holography for telemedicine applications. Lab. Chip 10, 1417 (2010). 20401422 10.1039/c000453g PMC2902728

[b42] Mudanyali O. . Wide-field optical detection of nanoparticles using on-chip microscopy and self-assembled nanolenses. Nat. Photonics 7, 247–254 (2013). 10.1038/nphoton.2012.337 PMC3866034 24358054

[b43] Hardie R. C., Barnard K. J., Bognar J. G., Armstrong E. E. & Watson E. A. High-resolution image reconstruction from a sequence of rotated and translated frames and its application to an infrared imaging system. Opt. Eng. 37, 247–260 (1998).

[b44] Elad M. & Hel-Or Y. A fast super-resolution reconstruction algorithm for pure translational motion and common space-invariant blur. IEEE Trans. Image Process. 10, 1187–1193 (2001). 18255535 10.1109/83.935034

[b45] Farsiu S., Elad M. & Milanfar P. Multiframe demosaicing and super-resolution of color images. IEEE Trans. Image Process. 15, 141–159 (2006). 16435545 10.1109/tip.2005.860336

[b46] Luo W., Zhang Y., Feizi A., Göröcs Z. & Ozcan A. Pixel super-resolution using wavelength scanning. Light Sci. Appl. 5, e16060 (2016). 10.1038/lsa.2016.60 PMC6059953 30167157

[b47] Luo W., Zhang Y., Göröcs Z., Feizi A. & Ozcan A. Propagation phasor approach for holographic image reconstruction. Sci. Rep. 6 (2016). 10.1038/srep22738 PMC4786813 26964671

[b48] Goodman J. W. Introduction to Fourier optics. (Roberts & Co 2005).

[b49] Bishara W. . Holographic pixel super-resolution in portable lensless on-chip microscopy using a fiber-optic array. Lab. Chip 11, 1276 (2011). 21365087 10.1039/c0lc00684j PMC3151573

[b50] Hecht E. Optics. (Addison-Wesley 2002).

[b51] Oppenheim A. V., Willsky A. S. & Nawab S. H. Signals & systems. (Prentice Hall 1997).

[b52] Memmolo P., Paturzo M., Javidi B., Netti P. A. & Ferraro P. Refocusing criterion via sparsity measurements in digital holography. Opt. Lett. 39, 4719 (2014). 25121857 10.1364/OL.39.004719

[b53] Allen L. J. & Oxley M. P. Phase retrieval from series of images obtained by defocus variation. Opt. Commun. 199, 65–75 (2001).

[b54] Reed Teague M. Deterministic phase retrieval: a Green’s function solution. J. Opt. Soc. Am. 73, 1434 (1983).

[b55] Waller L., Tian L. & Barbastathis G. Transport of Intensity phase-amplitude imaging with higher order intensity derivatives. Opt. Express 18, 12552–12561 (2010). 20588381 10.1364/OE.18.012552

[b56] Dieppe P. A. . Synovial Fluid Crystals. QJM 48, 533–553 (1979). 231795

[b57] Phelps P. Compensated Polarized Light Microscopy: Identification of Crystals in Synovial Fluids From Gout and Pseudogout. JAMA 203, 508 (1968). 5694150 10.1001/jama.203.7.508

[b58] Research System Microscope BX51/BX61 BX2 series. Available at: http://www.olympuslatinoamerica.com/spanish/seg/img/Catalog/BX51_BX61_new_catalog.pdf.

